# Supplementary material for: Impact of end-stage knee osteoarthritis on perceived physical function and quality of life: A descriptive study from Jordan
Source: PLoS One. 2023 Jun 9;18(6):e0286962. doi: 10.1371/journal.pone.0286962 (PMC10256207; doi:10.1371/journal.pone.0286962)
Supplement: Attachment — Submitted filename: Response to reviewers..docx [file pone.0286962.s002.docx]

**Dear Editor and reviewers,**

Thank you for your comments, and we greatly appreciated your constructive criticisms in evaluating our manuscript entitled “Impact of end-stage knee osteoarthritis on perceived physical function and quality of life in the Middle East: A descriptive study in Jordan”.

We have addressed the reviewers’ concerns and comments as outlined below. We have responded on a point-by-point basis to the reviewers’ comments below. Changes that were made to the manuscript are provided on this document highlighted in yellow and in italics, as well as highlighted in the updated manuscript.

We hope that our responses and the updated manuscript are satisfactory and sufficiently address your concerns about the study.

**ACADEMIC EDITOR:**

**Comment:** Revise according to the reviewers' comments and clearly highlight potential limitations for this study and future directions.

**Reply to editor’s comment:** We carefully revised our manuscript based on your and the reviewer's comments. Thank you for your consideration.

The revised potential limitations and future directions were highlighted in our revised manuscript as follows:

*“Even though knee OA is more prevalent among female patients, the overrepresentation of females in our study participants and the exclusion of comorbid conditions that are common in this age group may limit the generalizability of our findings to all individuals with knee OA. The effect of gender on KOOS outcome couldn’t be evaluated in this study due to the limited number of male participants, and this could be investigated in* *a future study with a more diverse and representative sample.”*

*“Future studies that investigate the influence of such variables on knee-related perceived function and quality of life, could provide a more comprehensive understanding of the subject, which could, in turn, inform the development of more effective interventions for patients with end-stage knee OA.”*

**Reviewer #1:**

**Nice work! I would suggest to mention about how the Knee Injury and Osteoarthritis Outcome Score (KOOS) was translated to Arabic. Having only 4 male out of 52 participants is not optimal. Also, I am not sure if you calculated the power analysis. There were no justification about why you used that normalization method for KOOS.**

Dear reviewer, thank you very much for your valuable feedback and comments to improve the quality of our paper. We tried to revise it as much as we could in line with your feedback.

**Comment #1:** I would suggest to mention about how the Knee Injury and Osteoarthritis Outcome Score (KOOS) was translated to Arabic.

**Reply to comment #1:** Based on your suggestion, the following information was added and highlighted in the revised manuscript as follows:

*“Alfadhel et al. (2018) translated and cross-culturally adapted the original KOOS questionnaire into Arabic in Saudi Arabic patients with knee OA. The psychometric properties of the KOOS's Arabic version were evaluated in that study, and the results showed that the Arabic version of the KOOS is well accepted and has excellent reliability, internal consistency, and construct validity.*

**Comment #2:** Having only 4 male out of 52 participants is not optimal.

**Comment #3:** Also, I am not sure if you calculated the power analysis.

**Reply to Comments #2&3:** Thank you for your comment regarding the gender distribution of our study participants and the power analysis.

We recognize that the low number of male participants is not ideal and may affect the generalizability of our findings. We made every effort to recruit as many male participants as possible, but we were only able to recruit four male participants out of the 52 participants. However, our participants in this sample were recruited from a specific population where the proportion of the proportion of females to males is high. This point was previously elaborated on in the discussion section: “Only four males took part in this study, which may reflect the fact that more women than men in Jordan have knee OA and are awaiting TKA. In a hospital-based cohort study in Jordan, women constitute 61.4% of patients diagnosed with severe radiographic knee OA [14]. Besides, in the USA, women are 40% more likely than men to develop knee osteoarthritis [18] and over 60% of TKA patients are females [8].”

In this study we did not originally plan to exclusively focus on female patients. As a result, the data from the four male participants was included. Moreover, we conducted the analysis with and without the male participants data and found that results remained unchanged **(Tables for the female data are provided below).** The effect of gender on KOOS outcome couldn’t be evaluated in this study due to the limited number of male participants, and this could be investigated in a future study that involves a larger number of participants.

Additionally, post hoc power analysis using g-power software with 4 predictors in the model, alpha equals 0.05 and partial R square equals 0.67 revealed that regression analysis is 100% statically powered with either 48 female participants or all 52 participants.

We have modified the statement in the result section justifying the inclusion of male participants in this analysis as follows:

*“**This study sample included 52 participants. We were only able to recruit four male participants out of the 52 participants, who were drawn from a population with a high female-to-male ratio. The data from the four male participants was included in this analysis, as we did not originally plan to exclusively focus on female patients. Additionally, we conducted the analysis with and without the male participants data and found that results remained unchanged.”*

We have also acknowledged this point in limitation section of our updated manuscript as follows:

*“Even though knee OA is more prevalent among female patients, the overrepresentation of females in our study participants and the exclusion of comorbid conditions that are common in this age group may limit the generalizability of our findings to all individuals with knee OA. The effect of gender on KOOS outcome couldn’t be evaluated in this study due to the limited number of male participants, and this could be investigated in* *a future study with a more diverse and representative sample.”*

**Comment #4:** There were no justification about why you used that normalization method for KOOS.

**Reply to comment #4:** We agree that it is important to provide a clear explanation for the choice of the normalization method. This method was used according to KOOS scoring guideline 2012 ([www.koos.nu](http://www.koos.nu)), this is the formula used to calculate the score for each subscale to have it out of 100. This method allows for easier interpretation of the results and facilitates comparisons across different studies. We have revised our manuscript to include an explanation of the rationale for the choice of normalization method as follows:

*“This normalization method is used in accordance with the KOOS scoring guideline 2012 (available at www.koos.nu), which calculates the total score as shown in equation 1.”*

| **Table1: Anthropometric characteristics, pain and KOOS scores (Only Females, N=48)** | | | |
| --- | --- | --- | --- |
|  | Mean | SD | Range |
| **Height (cm)** | 154.92 | 6.99 | 125-170 |
| **Weight (Kg)** | 81 | 13.73 | 41-129 |
| **BMI (Kg/m^2^)** | 33.70 | 5.19 | 23.4-53 |
| **Age (yrs.)** | 66.27 | 6.64 | 50-78 |
| **Knee Pain “most affected” (0-10)** | 8.01 | 1.75 | 3-10 |
| **Knee Pain “less affected” (0-10)** | 4.15 | 3.03 | 0-10 |
| **KOOS-symptom (in %) 77.1** | 54.18 | 20.90 | 10.71-92.86 |
| **KOOS-pain (%) 78.6** | 45.77 | 20.59 | 2.78-84.38 |
| **KOOS-ADL (%) 77.4** | 46.34 | 19.38 | 4.69-85.71 |
| **KOOS-QOL (%) 68.6** | 27.34 | 17.13 | 0-87.5 |

| **Table 3. Hierarchical linear regression analysis with KOOS-ADLS and KOOS-QOL as dependent variables, and age, BMI, knee pain as independent variables (only females)** | | | | | |
| --- | --- | --- | --- | --- | --- |
|  | R | R^2^ | R^2^ change | Significance of change | Significance of model |
| KOOS-ADLS  Model  1  2  3 | 0.286  0.551  0.893 | 0.082  0.254  0.777 | 0.082  0.222  0.493 | 0.160  0.001  0.000 | 0.160  0.002  0.000 |
| KOOS-QOL  Model  1  2  3 | 0.223  0.302  0.711 | 0.050  0.091  0.505 | 0.050  0.041  0.414 | 0.333  0.176  0.000 | 0.333  0.256  0.000 |
| * **Model 1**= body mass index (BMI) and age; **Model 2**= BMI and age, pain in the “less affected” knee; **Model 3**= BMI and age, pain in the” less affected” knee, and pain in the “most affected” knee | | | | | |

**Reviewer #2:**

**İn line 97-101** **Jordanians have cultural habits that involve cross-sitting and kneeling for lengthy periods during the day, most of them are Arab Muslims who pray five times a day, and this religious activity includes kneeling, knee bending, and frequent squatting which might exacerbate the loads on the knees. They also tend to postpone surgical intervention decisions.**

**( This sentence is just your opinion. Not well-intentioned also. The literature says the opposite. You haven't even looked at the literature. You can look this study,** **Yılmaz, S., Kart-Köseoglu, H., Guler, O., & Yucel, E. (2008). Effect of prayer on osteoarthritis and osteoporosis. Rheumatology International, 28, 429-436. )**

Thank you for your feedback on our manuscript. We appreciate your comments and have carefully considered your concerns. We have reviewed the literature, including the study you have suggested, and revised our manuscript accordingly.

Regarding our sentences in line 97-101, we acknowledge that these reflect our opinion that is supported by our observations and to some extent by evidence from literature.

We apologize for any misunderstanding. When we wrote this section (97-101), we were elaborating more on a point that was mentioned earlier on same paragraph regarding the need to conduct this study on Jordanian population “, the impact of knee OA may vary among different populations due to different lifestyles, everyday activities, and sociocultural differences”. Our intention was to provide specific examples of the daily activities in the Jordanian population that could impact how individuals with Knee OA in Jordan perceive their pain and functional abilities. While we acknowledge that Islamic Prayer is one of essential pillars in Islam and has been linked to positive effects on various health domains and well-being[1,2], it was mentioned in this context because it involves daily repetitive knee bending.

We understand the importance of supporting our opinion with relevant literature and have reviewed the literature on this topic. The literature shows inconsistent findings when it comes to the impact of prayers and floor activities on Knee OA. Yesil et al (2014) showed that regular prayer habit, among other factors, was found to be a risk factor for symptomatic knee OA[3]. Another study by Tangtrakulwanich and colleagues showed that habitual lifetime floor activities including squatting, and side-knee bending positions increase the risk of knee osteoarthritis[4], and frequent knee bending activities were associated with a higher prevalence of knee cartilage lesions in a study conducted by Virayavanich et al 2013[5]. On the other hand, a study by Yılmaz 2007 revealed that Prayer has no effect on knee and hip osteoarthritis[6]. Other studies showed that Islamic prayer may decrease the chances of development of knee osteoarthritis[7,8]. A study in Kurdistan region showed that knee OA is less prevalent in men who practice Islamic praying compared to who did not[7]. Similarly, a study conducted on Thai elderly Muslims and Buddhists found that prevalence of Knee OA was lower among the Muslim group compared to the Buddhist group[8].

Upon reviewing the literature and acknowledging the conflicting results on the effects of floor activities and prayer on Knee OA, we have decided to remove our proposed viewpoint to ensure the accuracy and validity of our manuscript.

**References for reviewer #2:**

1.	Chamsi-Pasha M, Chamsi-Pasha H. A review of the literature on the health benefits of salat (Islamic prayer). Medical Journal of Malaysia. 2021.

2.	Nazish N, Kalra N. Muslim Prayer-A New Form of Physical Activity: A Narrative Review. Int J Heal Sci Res. 2018.

3.	Yesil H, Hepguler S, Ozturk C, Yesil M, Capaci K. Risk Factors of Symptomatic Knee, Hand and Hip Osteoarthritis in a Suburban Area of İzmir City. Türkiye Fiz Tip ve Rehabil Derg. 2014. doi:10.5152/tftrd.2014.72593

4.	Tangtrakulwanich B, Chongsuvivatwong V, Geater AF. Habitual floor activities increase risk of knee osteoarthritis. Clin Orthop Relat Res. 2007. doi:10.1097/01.blo.0000238808.72164.1d

5.	Virayavanich W, Alizai H, Baum T, Nardo L, Nevitt MC, Lynch JA, et al. Association of frequent knee bending activity with focal knee lesions detected with 3T magnetic resonance imaging: Data from the osteoarthritis initiative. Arthritis Care Res. 2013. doi:10.1002/acr.22017

6.	Yılmaz S, Kart-Köseoglu H, Guler O, Yucel E. Effect of prayer on osteoarthritis and osteoporosis. Rheumatol Int. 2008. doi:10.1007/s00296-007-0466-7

7.	AL-DABBAGH TAHER Q., RASOOL MT, AL- BARZINJY NJ. ISLAMIC PRAYING AND OSTEOARTHRITIS CHANGES OF WEIGHT BEARING JOINTS. Duhok Med J . 2009.

8.	Chokkhanchitchai S, Tangarunsanti T, Jaovisidha S, Nantiruj K, Janwityanujit S. The effect of religious practice on the prevalence of knee osteoarthritis. Clin Rheumatol. 2010. doi:10.1007/s10067-009-1295-8

**Reviewer #3:**

We greatly appreciate your valuable feedback aimed at enhancing the quality of our article. We are going to incorporate your recommendations and revise it accordingly

**Comment #1:** Author need to revised the title as in the title it is written middle east, whereas study has only included the participants from only one hospital with sample of 52 only. So this can't be generalize to whole middle east.

**Reply to comment #1:** Dear reviewer, we agree with you. The participants were from 2 local hospitals. We mentioned the middle east in the title as Jordan is one of middle east countries. The title has been modified to:

*“Impact of end-stage knee osteoarthritis on perceived physical function and quality of life: A descriptive study from Jordan”*

**Comment #2:** It will be good to add end-stage knee OA in the short title.

**Reply to comment #2:** Thank you. This has been added.

**Comment #3**: It will be good to remove KOOS in the keyword or write the full term.

**Reply to comment #3:** Thank you for your suggestion. We added the full name of the questionnaire. However, it is very common to search for papers using the questionnaire's abbreviated name as a keyword. Therefore, we would also like to keep the abbreviation as we believe it may enhance the paper's accessibility in the event of publication.

**Comment #4:** see the referencing style.

**Reply to comment #4:** References revised according to format instructions.

**Comment #5:** Result: Line 198-200: Doesn't make clear sense. Write it clearly.

**Reply to comment #5:** Dear reviewer, we apologize for any confusion caused by the unclear lines. We have revised this part and clarified the intended meaning according to your and other reviewer’s comments and highlighted as follows:

*“This study sample included 52 participants. We were only able to recruit four male participants out of the 52 participants, who were drawn from a population with a high female-to-male ratio. The data from the four male participants was included in this analysis, as we did not originally plan to exclusively focus on female patients. Additionally, we conducted the analysis with and without the male participants data and found that results remained unchanged.”*

**Comment #6:** Limitation: The major limitation of the study is limited sample size and majority are female. So, the findings can't be generalize to larger population. Although OA knee is common among female population.

**Reply to comment #6:** We agree with you. We acknowledge that the predominantly female population of our study is a limitation and that the findings of our study may not be generalizable to all individuals with end-stage knee OA. According to your and other reviewer’s comments we added statements about this limitation and the needed future studies as follows:

*“Even though knee OA is more prevalent among female patients, the overrepresentation of females in our study participants and the exclusion of comorbid conditions that are common in this age group may limit the generalizability of our findings to all individuals with knee OA. The effect of gender on KOOS outcome couldn’t be evaluated in this study due to the limited number of male participants, and this could be investigated in* *a future study with a more diverse and representative sample.”*

According to your and other reviewer’s comment regarding the limited sample size and power analysis, post hoc power analysis using g-power software with 4 predictors in the model, alpha equals 0.05 and partial R square equals 0.67 revealed that regression analysis is 100% statically powered with either 48 female participants or all 52 participants.
